# The prognostic association of *SPAG5* gene expression in breast cancer patients with systematic therapy

**DOI:** 10.1186/s12885-019-6260-6

**Published:** 2019-11-05

**Authors:** Chenjing Zhu, Otilia Menyhart, Balázs Győrffy, Xia He

**Affiliations:** 1 Department of Radiation Oncology, Jiangsu Cancer Hospital & Jiangsu Institute of Cancer Research & The Affiliated Cancer Hospital of Nanjing Medical University, 42 Baiziting, Nanjing, 210009 Jiangsu China; 2 TTK Cancer Biomarker Research Group, Institute of Enzymology, Budapest, Hungary; 3 Second Department of Pediatrics, Semmelweis University, Budapest, H-1094 Hungary

**Keywords:** SPAG5, Prognosis, Breast cancer, Endocrine therapy, Chemotherapy

## Abstract

**Background:**

Despite much effort on the treatment of breast cancer over the decades, a great uncertainty regarding the appropriate molecular biomarkers and optimal therapeutic strategy still exists. This research was performed to analyze the association of SPAG5 gene expression with clinicopathological factors and survival outcomes.

**Methods:**

We used a breast cancer database including 5667 patients with a mean follow-up of 69 months. Kaplan-Meier survival analyses for relapse free survival (RFS), overall survival (OS), and distant metastasis-free survival (DMFS) were performed. In addition, ROC analysis was performed to validate SPAG5 as a prognostic candidate gene.

**Results:**

Mean *SPAG5* expression value was significantly higher with some clinicopathological factors that resulted in tumor promotion and progression, including poor differentiated type, HER2 positive or TP53 mutated breast cancer. Based on ROC-analysis SPAG 5 is a suitable prognostic marker of poor survival. In patients who received chemotherapy alone, *SPAG5* had only a moderate and not significant predictive impact on survival outcomes. However, in hormonal therapy, high *SPAG5* expression could strongly predict prognosis with detrimental RFS (HR = 1.57, 95% CI 1.2–2.06, *p* = 0.001), OS (HR = 2, 95% CI 1.05–3.8, *p* = 0.03) and DMFS (HR = 2.36, 95% CI 1.57–3.54, *p* <  0.001), respectively. In addition, *SPAG5* could only serve as a survival predictor in ER+, but not ER- breast cancer patients. Patients might also be at an increased risk of relapse despite being diagnosed with a lower grade cancer (well differentiated type).

**Conclusions:**

*SPAG5* could be used as an independent prognostic and predictive biomarker that might have clinical utility, especially in ER+ breast cancer patients who received hormonal therapy.

## Background

Breast cancer is one of the leading types of cancer in women which accounted for about 39,620 deaths among US women in 2013 [1]. Despite much effort on the treatment of breast cancer over the decades, a great uncertainty regarding the optimal therapeutic strategy, especially effective precision medicine for breast cancer still exists [2]. As only those individuals who harbor the appropriate molecular biomarkers are eligible for effective precision treatment [3], identification, stratification and evaluation of better prognostic/predictive markers are in great need [4]. Nowadays, breast cancer systemic treatment strategies are guided by molecular subtypes based on estrogen receptor (ER), progesterone receptor (PR) and epidermal growth factor receptor 2 (HER2) statuses [5], and clinically useful biomarkers are demanded in predicting a patient’s response and long-term outcomes. Some potential indicators have been found in the diagnosis and therapeutic monitoring of patients with breast cancer, such as SASH1, cystatin C and activin A [6–8].

Sperm-associated antigen 5 (*SPAG5*, also named DEEPEST, MAP126 or hMAP126), located on chromosome 17q11.2, was up-regulated in M-phase cells and played a vital role in cell mitosis and cell cycle checkpoint regulation [9]. By binding to microtubules, it regulated the timing of spindle organization as well as separation of sister chromatids [10]. In addition, *SPAG5* protected cells from apoptosis via the mTOR signaling pathway [9, 11]. Knockdown of *SPAG5* could significantly suppress proliferation and invasion of prostate cancer cells in vitro, along with inhibiting the growth and metastasis of tumor in vivo [10].

Previous studies indicated that the overexpression of *SPAG5* gene might act as a potential biomarker which predicted poor prognosis in patients with lung cancer and cervical cancer [11, 12]. However, few studies focused on the prognostic value of *SPAG5* in breast cancer patients*.* A recent study [13] reported that the transcript and protein products of *SPAG5* might be independent prognostic and predictive biomarkers for chemotherapy sensitivity, particularly in ER negative (ER-) breast cancer. One stated the prognostic association of SPAG5 in ER+ breast cancer [14]. In addition, SPAG5 module was found to be involved in the mitotic checkpoint and associated with proliferation and progression of male breast cancer (MBC) [15].

To comprehensively assess the association of SPAG5 gene expression with clinical outcomes in patients with different breast cancer subtypes, including those undergoing systematic treatment (endocrine therapy and/or chemotherapy), we used a large public database containing pure transcriptomic data of more than 5000 breast cancer patients and validated *SPAG5* as a prognostic candidate gene.

## Methods

### Breast cancer microarray database

Kaplan–Meier Plotter (http://www.kmplot.com) is an online public database evaluating the effect of 54,675 genes on patient clinical outcomes, using 10,293 samples of lung, breast, gastric or ovarian cancers. This online tool is handled by a PostgreSQL server that could simultaneously integrate gene expression and clinical data [16, 17]. Gene expression data and the survival information are derived from the Gene Expression Omnibus (GEO), The Cancer Genome Atlas (TCGA) and European Genome-phenome Atlas (EGA) (see Additional file 3: Table S1).

### Data retrieval

We performed data retrieval from the online tool from July 2016 to October 2016. The database contained information of 5667 patients with breast cancer, with a median follow-up of 69 months. It allowed for filtering by ER, PR and HER2 statuses, lymph node statuses (positive or negative), grade (I, II or III) and TP53 statuses (mutated or wild type) [18]. In addition, analyses could be restricted to cohorts that only included patients with endocrine treatment or chemotherapy. Biased arrays were excluded. Although not all clinic-pathological data and survival outcomes were obtainable in each patient, we reported all available data.

### Statistical analysis

We compared *SPAG5* gene expression level using Kruskal-Wallis test (multi-group comparisons) or Mann-Whitney U test (two-cohort comparison). Mean expression value, 95% Confidence Interval (CI), standard error and standard deviation were analyzed. For the prognostic value of gene *SPAG5*, we plotted the Kaplan–Meier curves for *SPAG5* (Affymetrix ID: 203145_at) in different breast cancer subtypes. The cutoff value of gene expression was chosen as median which split the patient samples into two groups and plots generated accordingly. The two patient cohorts were then compared, and we performed univariate Cox regression to calculate the hazard ratio (HR) with 95% confidence intervals (CIs) and log rank *P*-value. As not every patient’s data was included in the database that we needed to perform multiple Cox regression analyses, it was the best to do the multiple hypothesis testing [19, 20].

In addition, ROC analysis was performed by splitting the population into good and poor-outcome based on RFS, and we checked whether SPAG5 expression recognizes poor/good survival. We run the analysis for RFS of the entire dataset, ER-positive population and ER-positive population treated with endocrine therapy at 5 years and 10 years, respectively. Evaluation of gene *SPAG5* with relapse free survival (RFS), overall survival (OS) and distant metastasis-free survival (DMFS) was performed. We also used this Kaplan-Meier Plotter to stratify breast cancer patient microarray data by ER, PR, HER2, lymph node status, histological grade and TP53 status, and explored the prognostic value of *SPAG5* in those different breast cancer subtypes. We explored the survival of patients with different treatment strategies (hormonal therapy and/or chemotherapy). *P*-value <  0.05 was considered to be a statistically significant difference.

## Results

### *SPAG5* gene expression in breast cancer patients

The Kaplan–Meier Plotter surveyed public microarray data repositories for survival among 5667 patients with breast cancer. Mean *SPAG5* expression value was higher in ER- than ER+ breast cancer patients (mean value 434.48 vs. 602.64, *p* <  0.001), similar trend was also observed in PR- and HER2+ breast cancer patients. In addition, *SPAG5* expression was progressively higher in more aggressive grades/subtypes of the disease (see Additional file 3: Tables S2 and S3 and Additional file 1: Figure S1).

### *SPAG5* gene expression was associated with breast cancer progression and poor prognosis

**Fig. 1:**
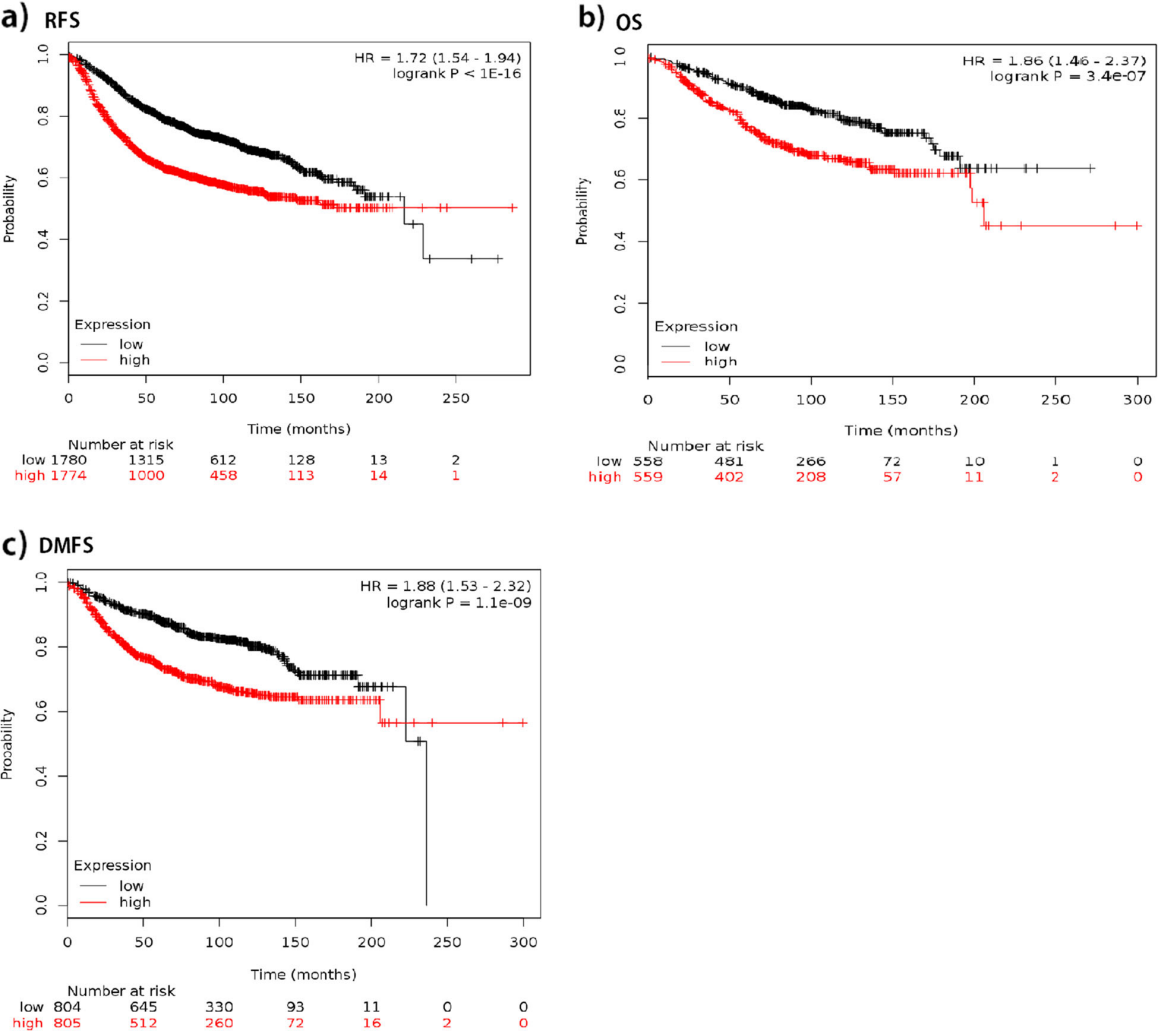
The prognostic effect of the expression of *SPAG5* in www.kmplot.com. **a** RFS **b** OS **c** DMFS

**Fig. 2:**
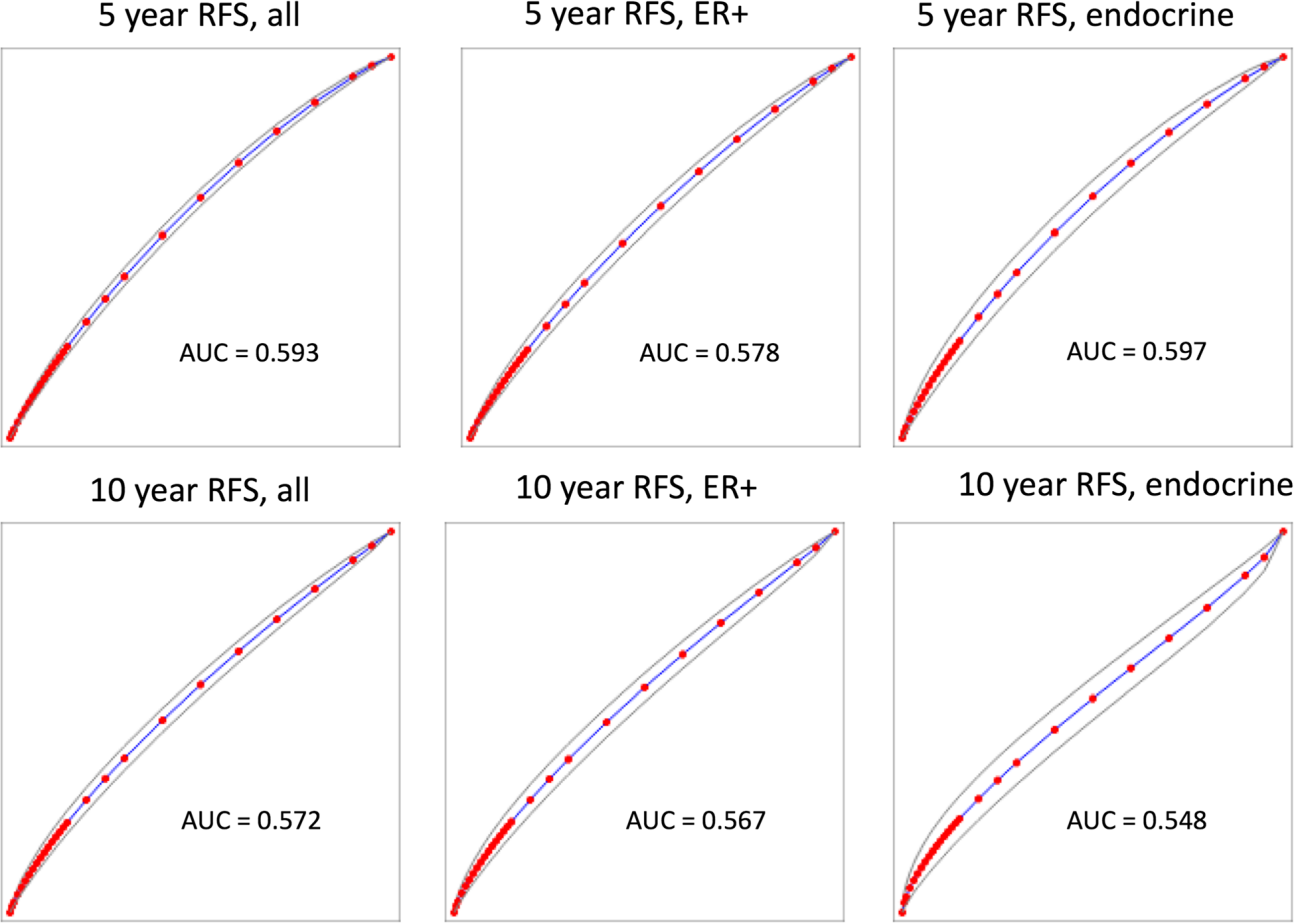
ROC analysis was performed by splitting the population into good and poor-outcome based on RFS, the analysis was run for RFS of the entire dataset, ER+ population and ER+ population treated with endocrine therapy at 5 years and 10 years, respectively

We plotted the Kaplan-Meier survival curves for *SPAG5* using the web-based curator. The results showed that higher expression of *SPAG5* was associated with worse RFS (*n* = 3557, HR = 1.72, 95% CI 1.54–1.94, *p <* 0.001), OS (*n* = 1117, HR = 1.86, 95% CI 1.46–2.37, *p <* 0.001), and DMFS (*n* = 1610, HR = 1.88, 95% CI 1.53–2.32, *p* < 0.001) in patients with breast cancer. Table 1 and Fig. 1 present the prognostic effect of the expression of *SPAG5*. In addition, we compared and correlated SPAG5 with other markers of progression, such as p53, AURKA, MKI67 and BIRC5, to assess independent value, and results showed that similar to AURKA, MKI67, BUB1, TOP2A which had statistically significant results for RFS, *SPAG5* was associated with breast cancer progression. There was a significant association (coefficient over 0.25, *p* < 0.001) of SPAG5 with TOP2A, BIRC5, AURKA and BUB1. The association with PCNA and TP53 was significant, but the effect was too small to be meaningful (Additional file 3: Table S4). Based on ROC analysis, SPAG5 is a suitable prognostic marker of poor survival (see Fig. 2).

**Table 1:** PH Cox regression univariate analyses for the association of gene *SPAG5* with cancer progression and prognosis in different breast cancer subtypes

Breast cancer subtypes	RFS			OS			DMFS		
	n	HR	*P*-value	n	HR	*P*-value	n	HR	*P*-value
Total	3557	1.72 (1.54–1.94)	< 0.001	1117	1.86 (1.46–2.37)	< 0.001	1610	1.88 (1.53–2.32)	< 0.001
ER status									
ER+	2766	1.77 (1.55–2.03)	< 0.001	377	2.74 (1.74–4.33)	< 0.001	577	2.89 (1.95–4.29)	< 0.001
ER-	788	1.03 (0.82–1.28)	0.81	142	0.91 (0.52–1.6)	0.74	170	1.04 (0.62–1.73)	0.89
PR status									
PR+	525	2.02 (1.38–2.94)	< 0.001	0	–	–	122	1.35 (0.41–4.46)	0.62
PR-	483	1.42 (1.04–1.93)	0.027	2	–	–	95	2.24 (1.04–4.85)	0.035
HER2 status									
HER2+	168	0.78 (0.46–1.32)	0.36	28	0.59 (0.19–1.83)	0.36	66	1.54 (0.61–3.91)	0.36
HER2-	756	1.78 (1.36–2.34)	< 0.001	62	0.92 (0.32–2.62)	0.87	82	2.43 (0.63–9.39)	0.18
ER+/PR+/HER2+	76	1.53 (0.33–7.09)	0.58	36	3.54 (0.41–30.58)	0.22	45	1.83 (0.36–9.47)	0.46
ER+/PR−/HER2+	26	0.47 (0.09–2.35)	0.35	–	–	–	–	–	–
ER+/PR+/HER2-	339	2.41 (1.48–3.93)	< 0.001	39	2.04 (0.18–22.51)	0.55	79	1.66 (0.33–8.22)	0.53
ER+/PR−/HER2-	77	1.47 (0.65–3.33)	0.35	–	–	–	–	–	–
ER−/PR−/HER2-	255	1.45 (0.9–2.34)	0.13	–	–	–	43	3.33 (0.67–16.58)	0.12
LN status									
LN+	945	1.63 (1.3–2.03)	< 0.001	197	1.38 (0.84–2.28)	0.2	337	1.74 (1.14–2.65)	0.009
LN-	1813	1.67 (1.4–1.99)	< 0.001	425	2.41 (1.56–3.74)	< 0.001	896	2.42 (1.79–3.27)	< 0.001
Grade									
1	308	2.52 (1.4–4.54)	0.0014	135	2.45 (0.86–6.96)	0.083	172	2.35 (0.94–5.84)	0.059
2	724	1.9 (1.45–2.49)	< 0.001	287	2.92 (1.76–4.86)	< 0.001	495	1.93 (1.34–2.78)	< 0.001
3	723	1.17 (0.91–1.51)	0.23	347	0.9 (0.6–1.34)	0.6	391	1.12 (0.77–1.64)	0.54
TP53 status									
Mutated	188	0.91 (0.57–1.47)	0.71	111	0.95 (0.45–2.04)	0.91	83	0.8 (0.33–1.93)	0.62
Wild type	273	1.49 (0.97–2.28)	0.064	187	2.16 (1.1–4.23)	0.022	109	3.44 (1.44–8.22)	0.0031

*RFS* Relapse free survival, *OS* Overall survival, *DMFS* Distant metastasis-free survival, *HR* Hazard ratio, − Ddata not available, *LN* Lymph node

### The expression of gene *SPAG5* in patients receiving systematic therapy

**Fig. 3:**
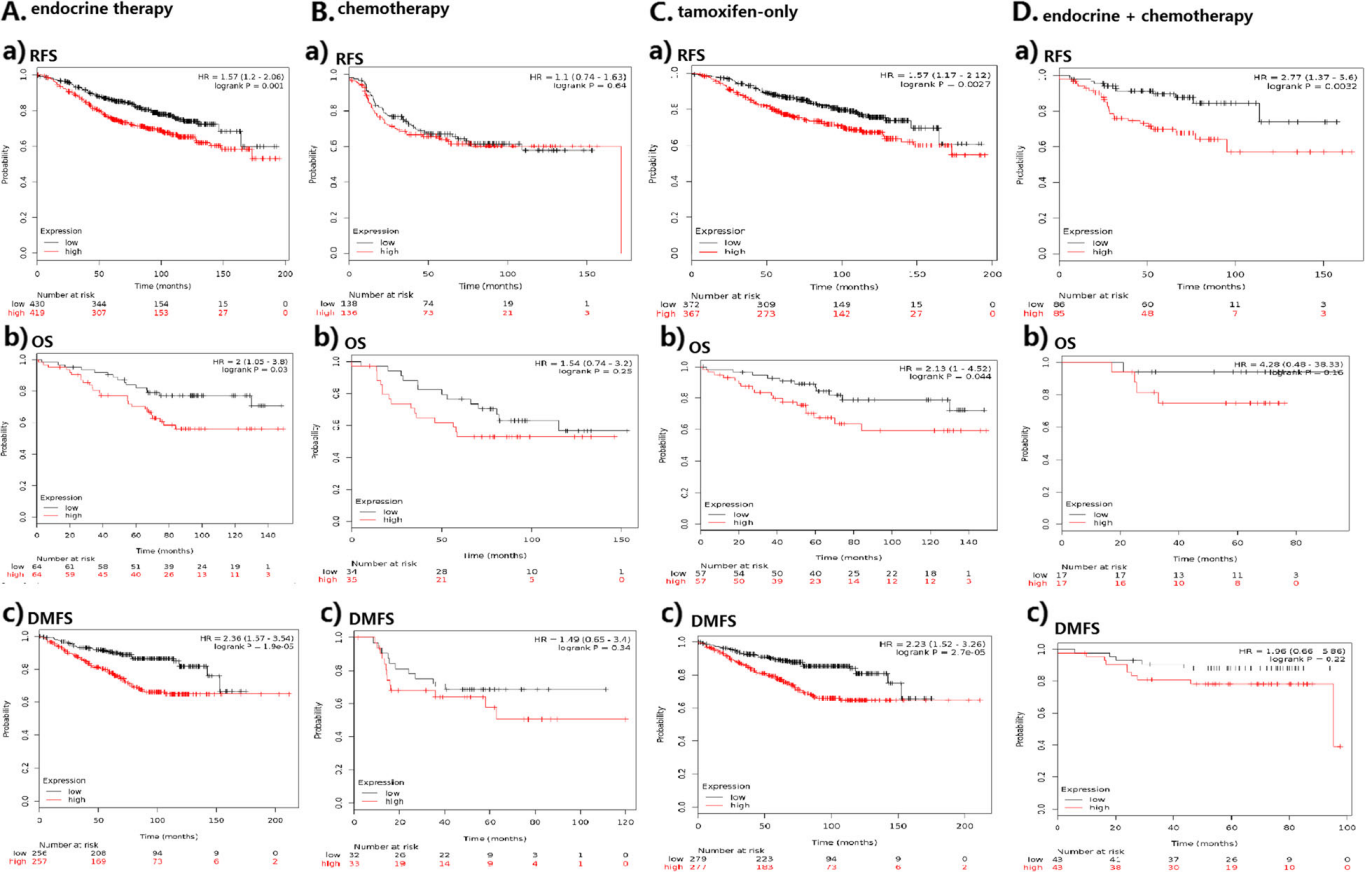
*SPAG5* in patients with systematic therapy in univariate analysis. **a**. Association of *SPAG5* with survival outcomes in patients with endocrine therapy. **b**. Association of *SPAG5* with survival outcomes in patients with chemotherapy. **c**. *SPAG5* expression was predictive of relapse in patients with tamoxifen-only therapy. **d**. *SPAG5* expression and survival outcomes in patients with both endocrine therapy and chemotherapy

In patients with endocrine therapy, low *SPAG5* transcript expression was significantly associated with longer RFS (HR = 1.57, 95% CI 1.2–2.06, *p* = 0.001) and OS (HR 2, 95% CI 1.05–3.8, *p* = 0.03) than was high *SPAG5* expression. However, in patients who received chemotherapy, no significant difference existed between low and high SPAG5 transcript expressions in RFS (HR = 1.1, 95% CI 0.74–1.63, *p* = 0.64) and OS (HR = 1.54, 95% CI 0.74–3.2, *p =* 0.25) of breast cancer patients. Similar results were also seen for DMFS in patients with endocrine therapy (HR = 2.36, 95% CI 1. 57–3.54, *p* < 0.001) and chemotherapy (HR = 1.49, 95% CI 0.65–3.4, *p* = 0.34) (see Table 2 and Fig. 3). Tamoxifen was the most common drug used in hormonal therapy, and in patients receiving tamoxifen-only therapy, *SPAG5* overexpression was associated with decreased RFS (HR = 1.57, 95% CI 1.17–2.12, *p* = 0.0027), OS (HR = 2.13, 95% CI 1.00–4.52, *p* = 0.044) and DMFS (HR = 2.23, 95% CI 1.52–3.26, *p* < 0.001). In 171 patients receiving both hormonal therapy and chemotherapy, *SPAG5* overexpression was associated with decreased RFS (HR = 2.77, 95% CI 1.37–5.6, *p* = 0.0032) and data for OS and DMFS among those patients were not enough to draw a concrete conclusion (see Table 2 and Fig. 3).

**Table 2:** PH Cox regression univariate analyses for the association of gene *SPAG5* with endocrine therapy and chemotherapy

	RFS			OS			DMFS		
	n	HR	*P*-value	n	HR	*P*-value	n	HR	*P*-value
Systemic therapy subtypes									
Endo	849	1.57 (1.2–2.06)	0.001	128	2 (1.05–3.8)	0.03	513	2.36 (1.57–3.54)	< 0.001
Tamoxifen-only	739	1.57 (1.17–2.12)	0.0027	114	2.13 (1–4.52)	0.044	556	2.23 (1.52–3.26)	< 0.001
Chemo	274	1.1 (0.74–1.63)	0.64	69	1.54 (0.74–3.2)	0.25	65	1.49 (0.65–3.4)	0.34
Endo + chemo	171	2.77 (1.37–5.6)	0.0032	34	4.28 (0.48–38.33)	0.16	86	1.96 (0.66–5.86)	0.22

*Endo* Endocrine therapy, *chemo* Chemotherapy

**Fig. 4:**
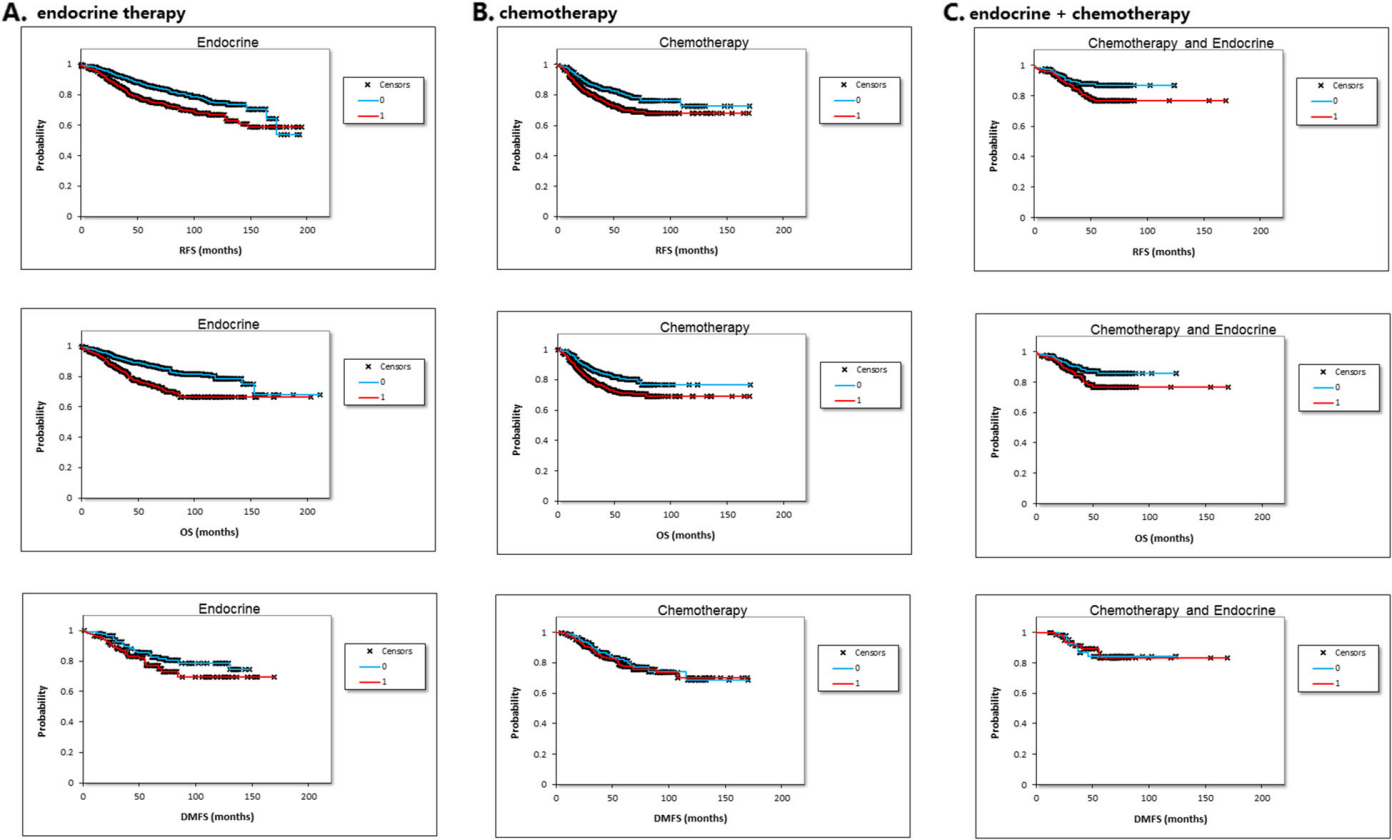
*SPAG5* in patients with systematic therapy in multiple hypothesis testing. **a** Association of *SPAG5* with survival outcomes in patients with endocrine therapy. **b** Association of *SPAG5* with survival outcomes in patients with chemotherapy. **c** *SPAG5* expression and survival outcomes in patients with both endocrine therapy and chemotherapy

In multiple hypothesis testing, the association remained significant in patients with endocrine therapy with poor RFS (HR = 1.61, 95% CI 1.26–2.04, *p* < 0.001) and OS (HR = 1.95, 95% CI 1.47–2.60, *p* < 0.001). Data for DMFS and tamoxifen-only therapy was not enough for multivariate analysis (Table 3 and Fig. 4).

**Table 3:** Multiple hypothesis testing of the association of gene *SPAG5* with endocrine therapy and chemotherapy

	RFS			OS			DMFS		
	HR	95% CI	*P*-value	HR	95% CI	*P*-value	HR	95% CI	*P*-value
Systemic therapy subtypes									
Endocrine therapy	1.61	1.26–2.04	< 0.001	1.95	1.47–2.60	< 0.001	1.45	0.85–2.48	ns,0.17
Chemotherapy	1.54	1.21–1.98	< 0.001	1.57	1.16–2.12	0.0033	1.10	0.70–1.71	ns,0.68
Endo + chemo	1.69	1.02–2.82	ns,0.0428	1.67	1.01–2.80	ns,0.051	0.99	0.34–2.86	ns,0.988

*ns* Not significant after correction for multiple hypothesis testing, *endo* Endocrine therapy, *chemo* Chemotherapy

### The prognostic value of *SPAG5* expression in breast cancer with different molecular subtypes, histological grades and TP53 statuses

When patients were differentiated based on ER expression statuses, we plotted RFS, OS and DMFS curves for the ER+ and ER- subsets. We observed that high *SPAG5* expression was associated with a significant increase in risk of relapse among ER+ (HR = 1.77, 95% CI 1.55–2.03, *p* < 0.001), but not ER- breast cancer patients (HR = 1.03, 95% CI 0.82–1.28, *p* = 0.81). Similarly, *SPAG5* gain or amplification was associated with shorter OS (HR = 2.74, 95% CI 1.74–4.33, *p* < 0.001) and DMFS (HR = 2.89, 95% CI 1.95–4.29, *p* < 0.001) in the ER+ subgroup, but not ER- subgroup (*p* = 0.74 and *p* = 0.89, respectively) (see Table 1 and Additional file 2: Figure S2). Likewise, in multiple hypothesis testing, higher expression of *SPAG5* was not associated with poorer survival in ER- subgroup, but the association was significant in ER+ patients with a reduction in RFS (HR = 1.85), OS (HR = 2.61) and DMFS (HR = 2.92) (see Table 4). In ER+/PR+/HER2- subgroup, *SPAG5* expression was associated with shorter RFS (HR = 2.41, 95% CI 1.48–3.93, *p* < 0.001) (see Table 1). We further stratified ER+ patients according to PR, HER2, lymph node status, histological grade and TP53 statuses and the results were listed in Additional file 3: Table S5.

**Table 4:** Multiple hypothesis testing of factors associated with survival

Breast cancer subtypes	RFS			OS			DMFS		
	HR	95% CI	*P*-value	HR	95% CI	*P*-value	HR	95% CI	*P*-value
ER status									
ER+	1.85	1.57–2.18	**< 0.001**	2.61	1.86–3.68	**< 0.001**	2.92	2.09–4.07	**< 0.001**
ER-	1.18	0.9–1.18	0.23	1.24	0.8–1.92	0.34	1.4	0.9–2.17	0.13
PR status									
PR+	3.4	2.09–5.55	**< 0.001**	6.34	1.26–31.88	ns, 0.011	3.92	1.15–13.32	ns, 0.018
PR-	1.51	1.11–2.05	ns, 0.0081	2.79	0.99–7.82	ns, 0.042	3.39	1.88–6.11	**< 0.001**
HER2 status									
HER2+	1.73	1.17–2.56	ns, 0.0053	3.42	1.52–7.72	**0.0017**	1.78	0.94–3.39	ns, 0.073
HER2-	2.12	1.79–2.51	**< 0.001**	1.7	1.37–2.12	**< 0.001**	1.93	1.58–2.36	**< 0.001**
Lymph node status									
Lymph node+	2.19	1.67–2.88	**< 0.001**	1.72	1.18–2.5	ns, 0.0044	2.03	1.4–2.94	**< 0.001**
Lymph node-	1.82	1.52–2.17	**< 0.001**	3	1.88–4.78	**< 0.001**	2.39	1.8–3.19	**< 0.001**
Grade									
1	3.12	1.8–5.42	**< 0.001**	3.19	1.28–7.96	ns, 0.0087	2.79	1.23–6.33	ns, 0.011
2	1.99	1.56–2.55	**< 0.001**	2.55	1.67–3.89	**< 0.001**	2.61	1.85–3.68	**< 0.001**
3	1.35	1.07–1.7	ns, 0.011	1.25	0.89–1.77	0.19	1.75	1.13–2.73	ns, 0.012
TP53 status									
Mutated	0.77	0.48–1.26	0.3	0.69	0.34–1.42	0.32	0.56	0.26–1.22	0.14
Wild type	1.82	1.16–2.85	ns, 0.0077	2.56	1.38–4.76	**0.0021**	3.67	1.76–7.63	**< 0.001**

*ns* Not significant after correction for multiple hypothesis testing; bold faced: remained significant

**Fig. 5:**
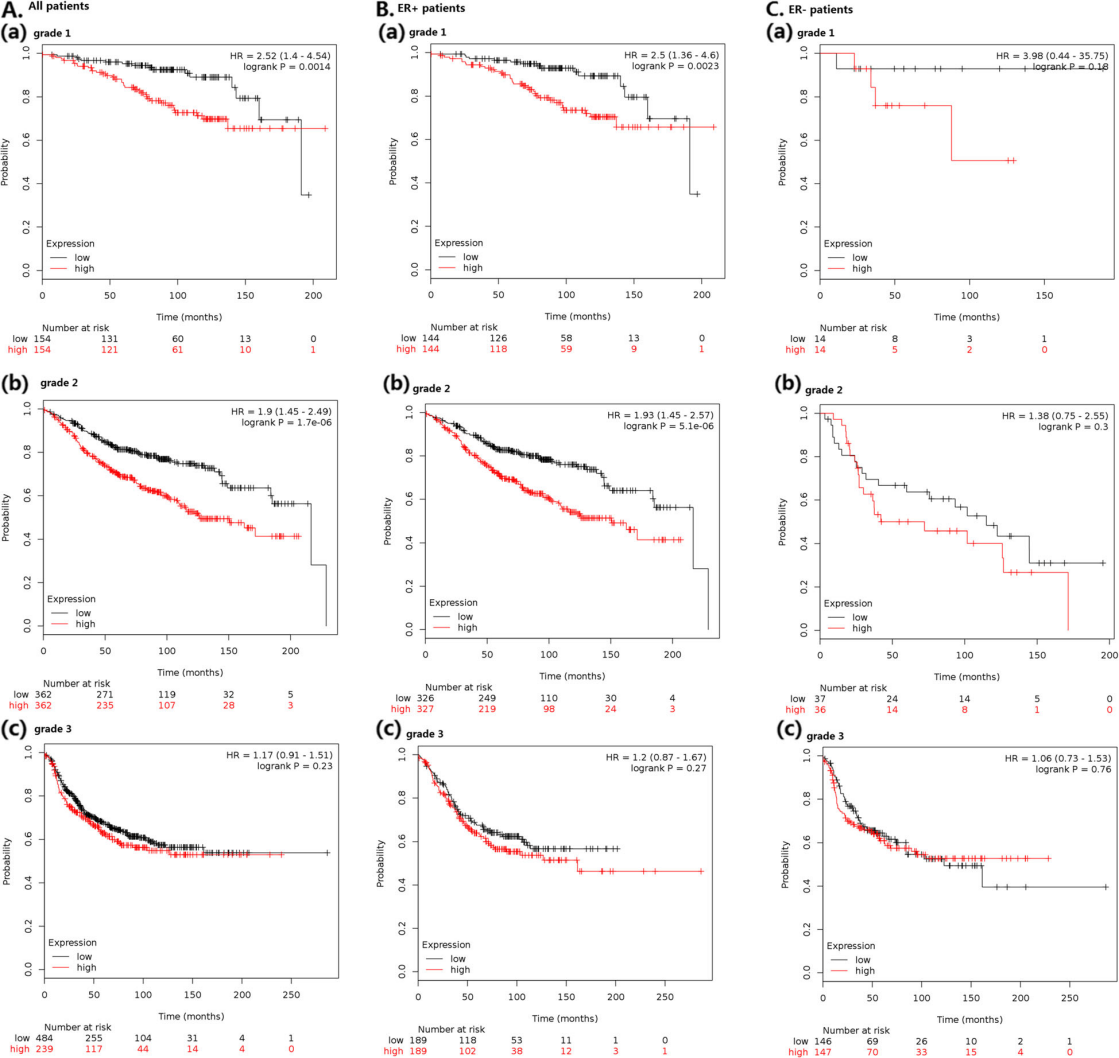
Expression of *SPAG5* with RFS among A. all patients; B. ER+ breast cancer patients; C. ER- patients with grade 1–3 breast cancers

Among patients with grade 1 breast cancer, high *SPAG5* expression was associated with a great increase in risk of recurrence (HR = 2.52, 95% CI 1.4–4.54, *p* = 0.0014). In more advanced cancers, high expression of *SPAG5* indicated less of an association with RFS in grade 2 cancer (HR = 1.9, 95% CI 1.45–2.49, *p* < 0.001), and only a moderate tendency with no statistical difference toward shorter RFS was seen among patients with grade 3 cancer (HR = 1.17, 95% CI 0.91–1.51, *p* = 0.23) (see Table 1 and Fig. 5). In TP53 wild-type breast carcinomas, RFS (HR = 1.49, 95% CI 0.97–2.28, *p* = 0.064), OS (HR = 2.16, 95% CI 1.1–4.23, *p* = 0.022) and DMFS (HR = 3.44, 95% CI 1.44–8.22, *p* = 0.0031) were better in patients with low-expressed *SPAG5*, however, the survival curves did not show a significant difference in RFS of TP53-mutated breast cancer patients (*p* = 0.71). RFS was low in HER2- patients (HR = 1.78, 95% CI 1.36–2.34, *p* < 0.001), but this prognostic association was not obvious in HER2+ patients (HR = 0.78, 95% CI 0.46–1.32, *p* = 0.36). Results of *SPAG5* expression in different PR statuses and lymph node statuses were also exhibited in Table 1. Multiple hypothesis testing supported the prognostic association of *SPAG5* in these different subgroups of patients (see Table 4).

## Discussion

In recent years, more and more attention has been attached on precision medicine, and there is a growing need for identification of prognostic biomarkers. *SPAG5*, originally identified as a microtubule-associated protein, with dual centrosome and kinetochores localization [21], has been reported to act as a promoter in tumorigenesis and progression [12]. In our study, data mining of 5667 publically available gene expression microarrays showed that elevated *SPAG5* expression in breast cancer predicted a poor prognosis by the Kaplan-Meier method. We found that high *SPAG5* expression was associated with lower RFS, OS, and DMFS, and *SPAG5* might act as an important marker in systematic therapy, especially in ER+ breast cancer patients who received hormonal therapy.

*SPAG5* was reported to be up-regulated in M-phase cells and play a vital role in cell mitosis and cell cycle checkpoint regulation [9]. In previous studies, *SPAG5* was found increasing in many tumors and considered as a predominant oncogene in tumor promotion and metastasis [2]. Therefore, the identification of patients with higher *SPAG5* expression before treatment would be important for personalized treatment. In our study, using a large combined cohort, we demonstrated that *SPAG5* expression was significantly higher in patients with hormone negative (ER- and PR-) breast cancer. Meanwhile, we found that *SPAG5* expression was increased in HER2 positive, poor differentiated, lymph node positive and TP53 mutated breast cancer subtypes all of which were strongly associated with tumor progression. Since the oncogenic potential of *SPAG5* was also reported in prostate cancer [10], we hypothesized that *SPAG5* could serve as a marker in predicting breast cancer proliferation and progression.

Systemic therapy for patients with early-stage breast cancer (ie, stages IA, IB, IIA, IIB, and IIIA) included chemotherapy, endocrine therapy, and targeted therapy [22]. It was important to choose certain biomarkers that could predict response to therapy and clinical outcomes. Recently a research team applied an artificial neural network performing data mining functions on *SPAG5* and found that *SPAG5* expression products were independent predictors for response to chemotherapy in breast cancer [13]. Similarly, we found that *SPAG5* could predict prognosis of breast cancer patients with systemic treatment. However, our results suggested that in patients who received chemotherapy, *SPAG5* had a moderate impact on survival outcomes including RFS (HR = 1.1), OS (HR = 1.54) and DMFS (HR = 1.49) in univariate analysis and the survival curves did not show a significant difference. As was referred in Hayes’s study [23], a HR of less than 2 meant that the clinical value was uncertain. A previous research suggested that *SPAG5* could affect chemotherapy sensitivity of taxol in cell lines [2]. The causes of the inconsistency might be attributed to the different chemotherapy regimens and varying methodological qualities.

Endocrine therapy abrogating estrogen dependent cell proliferation has been shown to reduce recurrence and death [24] for most patients with ER+ breast cancer. Tamoxifen is a Selective Estrogen Receptor Modulator (SERM) widely used for adjuvant therapy [25] and could reduce 15-year risks of breast cancer recurrence and mortality rates after surgery [26] in ER+ breast cancer patients [27]. However, resistance to tamoxifen is frequent, and patients receiving adjuvant tamoxifen may eventually suffer recurrence or progression or even death from metastases [28]. We found that when patients received both hormonal therapy and chemotherapy, high *SPAG5* expression could predict poor prognosis with HRs for RFS, OS and DMFS of 2.77, 4.28 and 1.96, respectively, although for OS and DMFS the difference was not statistically significant. Therefore, we assumed that high *SPAG5* expression in breast cancer was potentially more relevant to malignant prognosis in hormonal therapy. Further, in hormonal therapy only, patients with high *SPAG5* expression suffered decreased RFS, OS and DMFS in both univariate and multiple hypothesis testing. We considered that *SPAG5* was correlated with mTOR signaling pathway activity during breast cancer treatment [2], and the cross-talk between the estrogen receptor and mTOR signaling pathway, the most well-known mechanism of endocrine resistance, led to poor prognosis of patients [29]. Therefore, *SPAG5* contributed to the development of hormonal therapy resistance in ER+ breast cancer and the expression level was predictive on the survival outcomes of patients undergoing endocrine therapy. Further laboratory studies and clinical trials are needed to fully establish the association of *SPAG5* in endocrine and tamoxifen-based therapy.

Choosing biomarkers based on different breast cancer subtypes to predict survival is vital for both doctors and patients. In clinical practice, ER, PR and HER2 statuses are biologic markers considered to be crucial factors for treatment [30]. In our study, the large cohort with 2766 samples proved apparent statistically significant difference between *SPAG5*-high and *SPAG5*-low expressions in ER+, but not ER- breast cancer, meaning that the expression level of *SPAG5* could serve as a survival predictor in ER+ rather than ER- breast cancer patients. It might be because almost all ER+ patients received hormonal therapy and *SPAG5* expression predicted survival of patients in hormonal therapy. In some breast cancer subtypes like PR+/ER+ breast cancer, positive *SPAG5* expression presented a strong trend toward being associated with lower RFS (Additional file 3: Table S5). Moreover, *SPAG5* was an important determinant of survival in HER2 negative rather than HER2 positive breast cancer patients.

Also in our study, RFS, OS and DMFS were better in TP53 wild-type breast carcinomas patients with low-expressed *SPAG5*, while the survival curves did not show a significant difference in the survival outcomes of TP53-mutated breast cancer patients. As mutations in TP53 might lead to overexpression of *SPAG5*, which was essential for promoting and regulating several aspects of mitosis, such as inactivating Separase which maintained the cohesion of sister chromatids, stabilizing mitotic spindle, enhancing the fidelity of chromosome segregation, and silencing spindle assembly checkpoint [31], G2/M phase transition and permanent cell cycling [32] could be triggered. Studies have reported that mutant TP53 was strongly associated with endocrine therapy resistance and agents dramatically increasing wild-type p53 levels could induce cell cycle arrest and apoptosis in cancer cells [33]. All these were in accordance with our hypothesis described previously that *SPAG5* was related to the development of hormone resistance in breast cancer.

Histological grade is an important factor that affected the prognosis in breast cancer. In our study, we found that *SPAG5* expression was not predictive in high grade (poorly differentiated) breast cancer patients, perhaps because poorly differentiated breast cancer cells proliferated fast and had a poor response to all kinds of therapies including hormonal therapy [34]. On the contrary, expression was strongly associated with survival outcomes in low histological grade/proliferative status. As is already known, *SPAG5* is associated with cell cycle progression and formation of malignancies [10]. Considering the function of *SPAG5* in progression of mitosis [35], these results might imply that early in the etiology of ER+ breast cancer subtypes, *SPAG5* contributed to disease progression [36]. The gradual loss of this effect might be caused by the activation of parallel oncogenic pathways [37], and therefore weakened the influence of *SPAG5* [38]*.*

The potential of *SPAG5* as a therapeutic target of breast cancer has been highlighted in some experiments. Down-regulation of *SPAG5* exerted an antitumor effect. A study indicated that when silencing the expression of *SPAG5* protein with RNA interference, multipolar and highly disorganized spindles were formed, inducing mitotic arrest [31] and apoptosis [39] through cell cycle deregulation and mitotic catastrophe. In cervical cancer cell lines, *SPAG5* down-regulation resulted in inhibition of cell growth and proliferation by inducing G2/M phase cell cycle arrest [40]. What’s more, due to the loss of microtubule-binding ability of *SPAG5*, suppression of cell migration and invasion also occurred [41]. Thus, in addition to a potential prognostic biomarker, *SPAG5* might act as a therapeutic target for breast cancer.

To our knowledge, this is the largest up-to-date research on the prognostic association of SPAG5 in different subtypes of breast cancer. We analyzed different subtypes of breast cancer comprehensively (including poor differentiated type, HER2 positive or TP53 mutated breast cancer), which was not reported previously. Our work presented that for chemotherapy, the survival of patients did not show a significant difference between low and high SPAG5 transcript expressions, but the prognostic association of SPAG5 in endocrine therapy and tamoxifen-only therapy was explored. We offered the potential to discriminate ER+ breast cancer patients at higher risks of relapse, as well as providing opportunities to customize therapies.

Our work has limitations. First, the molecular mechanism and association of *SPAG5* in tumorigenesis and progression have not yet been fully identified. Second, the data of survival outcomes of new drugs for ER+ breast cancers including palbociclib [42] were lacking. Third, the optimal cutoff points of *SPAG5* for survival prediction in breast cancer patients still merit further investigation. Therefore, further researches on the role of *SPAG5* in breast cancer are mandatory in the future.

## Conclusions

In conclusion, as a progression-driving oncogene, *SPAG5* was closely related to disease progression and malignant prognosis of ER+ breast cancer patients undergoing endocrine therapy, and might act as a therapeutic target for breast cancer.

## Supplementary information

**Additional file 1: Figure S1.** *SPAG5* gene expression in all breast cancer patients with different subtypes.

**Additional file 2: Figure S2.** Survival curves for the ER+ and ER- breast cancer subset. A. ER+ breast cancer patients; B. ER- breast cancer patients.

**Additional file 3: Table S1.** Datasets used for the analysis. **Table S2.** *SPAG5* expression in all breast cancer patients with different subtypes. **Table S3.** The comparison of gene expression level using Mann-Whitney U test or Kruskal-Wallis test. **Table S4.** The comparison and correlation of SPAG5 with other markers of progression in assessing independent value. **Table S5.** Subgroup analyses of *SPAG5* gene in association with RFS in ER+/- breast cancer subtype.

## Data Availability

The datasets generated and/or analyzed during the current study are available in the Kaplan–Meier Plotter (http://www.kmplot.com). All data generated or analyzed during this study are included in this published article and its supplementary information files.

## Abbreviations

DMFS: Distant metastasis-free survival
ER-: ER negative
ER: Estrogen receptor
ER+: ER positive
HER2: Epidermal growth factor receptor 2
HR: Hazard ratio
OS: Overall survival
PR: Progesterone receptor
RFS: Relapse free survival
*SPAG5*: Sperm-associated antigen 5
