# Supplementary figures and images for: The prognostic association of SPAG5 gene expression in breast cancer patients with systematic therapy
Source: BMC Cancer. 2019 Nov 5;19:1046. doi: 10.1186/s12885-019-6260-6 (PMC6833211; doi:10.1186/s12885-019-6260-6)

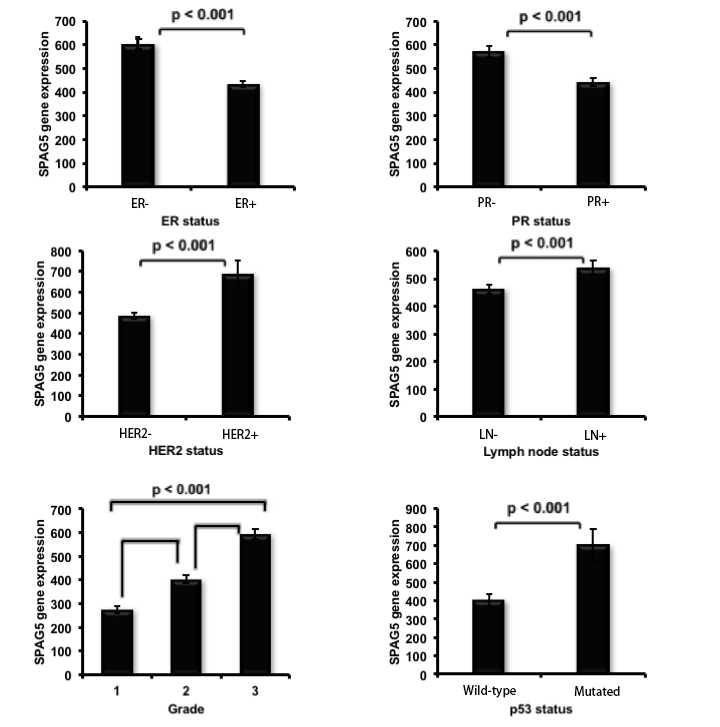

Supplement: Supplementary file 1 — Additional file 1: Figure S1. SPAG5 gene expression in all breast cancer patients with different subtypes. [file 12885_2019_6260_MOESM1_ESM.tiff]

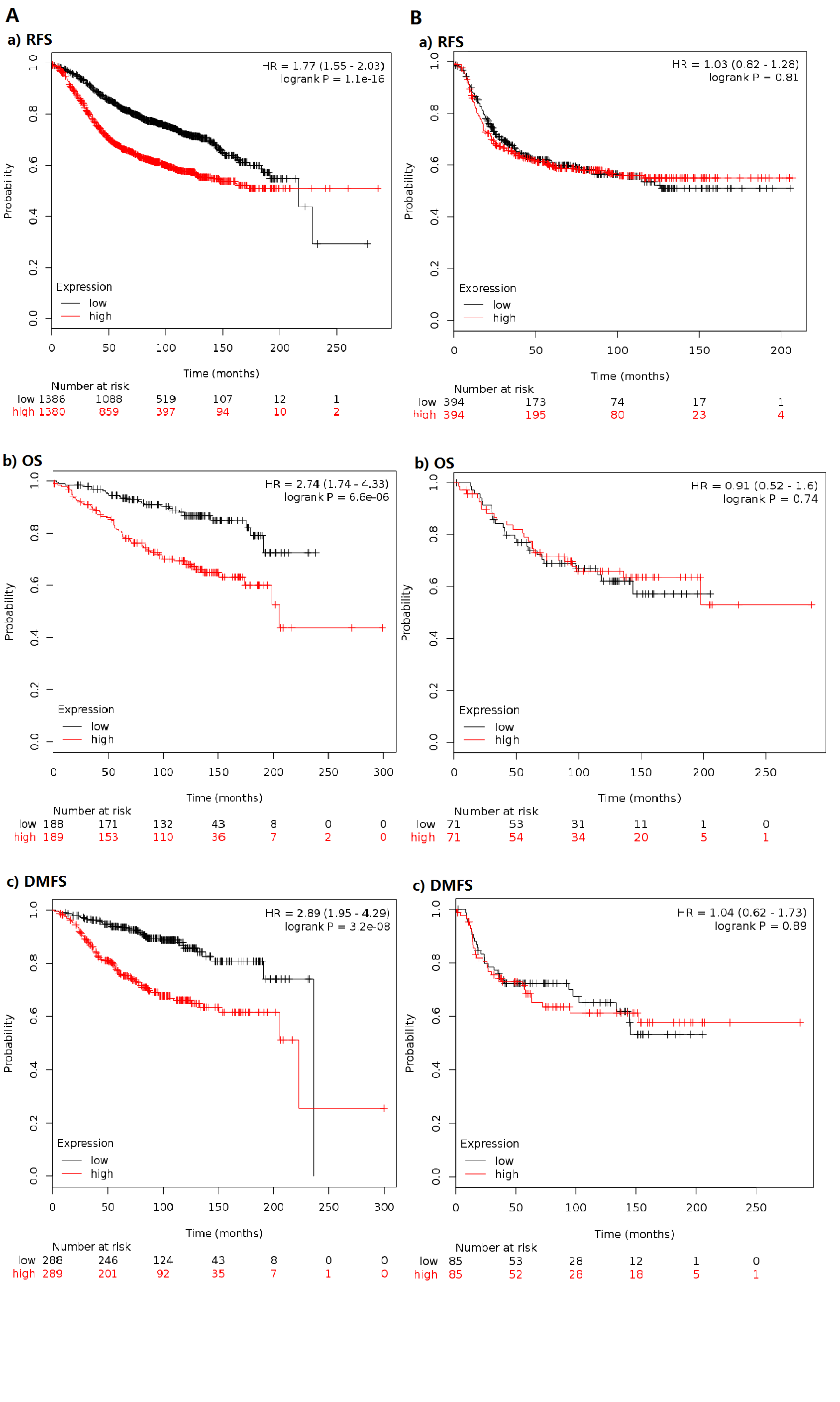

Supplement: Supplementary file 2 — Additional file 2: Figure S2. Survival curves for the ER+ and ER- breast cancer subset. A. ER+ breast cancer patients; B. ER- breast cancer patients. [file 12885_2019_6260_MOESM2_ESM.tiff]
