# Supplementary material for: The prognostic association of SPAG5 gene expression in breast cancer patients with systematic therapy
Source: BMC Cancer. 2019 Nov 5;19:1046. doi: 10.1186/s12885-019-6260-6 (PMC6833211; doi:10.1186/s12885-019-6260-6)
Supplement: Supplementary file 3 — Additional file 3: Table S1. Datasets used for the analysis. Table S2. SPAG5 expression in all breast cancer patients with different subtypes. Table S3. The comparison of gene expression level using Mann-Whitney U test or Kruskal-Wallis test. Table S4. The comparison and correlation of SPAG5 with other markers of progression in assessing independent value. Table S5. Subgroup analyses of SPAG5 gene in association with RFS in ER+/- breast cancer subtype. [file 12885_2019_6260_MOESM3_ESM.docx]

Table S1. Datasets used for the analysis.

| Dataset | Sample size | Platform | Reference |
| --- | --- | --- | --- |
| E-MTAB-365 | 537 | GPL570 | PMID:21785460 |
| E-TABM-43 | 37 | GPL96 | PMC1831731 |
| GSE11121 | 200 | GPL96 | [PMID: 18593943](https://www.ncbi.nlm.nih.gov/pubmed/18593943) |
| GSE12093 | 136 | GPL96 | [PMID: 18821012](https://www.ncbi.nlm.nih.gov/pubmed/18821012) |
| GSE12276 | 204 | GPL570 | [PMID: 19421193](https://www.ncbi.nlm.nih.gov/pubmed/19421193) |
| GSE1456 | 159 | GPL96, GPL97 | [PMID: 16280042](https://www.ncbi.nlm.nih.gov/pubmed/16280042) |
| GSE16391 | 55 | GPL570 | [PMID: 19573224](https://www.ncbi.nlm.nih.gov/pubmed/19573224) |
| GSE16446 | 120 | GPL570 | [PMID: 20098429](https://www.ncbi.nlm.nih.gov/pubmed/20098429) |
| GSE16716 | 47 | GPL96, GPL570 | [PMID: 20064235](https://www.ncbi.nlm.nih.gov/pubmed/20064235) |
| GSE17705 | 196 | GPL96 | [PMID: 20697068](https://www.ncbi.nlm.nih.gov/pubmed/20697068) |
| GSE17907 | 54 | GPL570, GPL9128 | [PMID: 20932292](https://www.ncbi.nlm.nih.gov/pubmed/20932292) |
| GSE18728 | 61 | GPL570 | [PMID: 20012355](https://www.ncbi.nlm.nih.gov/pubmed/20012355) |
| GSE19615 | 115 | GPL570 | [PMID: 20098429](https://www.ncbi.nlm.nih.gov/pubmed/20098429) |
| GSE20194 | 45 | GPL96 | [PMID: 20064235](https://www.ncbi.nlm.nih.gov/pubmed/20064235) |
| GSE20271 | 96 | GPL96 | [PMID: 20829329](https://www.ncbi.nlm.nih.gov/pubmed/20829329) |
| GSE2034 | 286 | GPL96 | [PMID: 15721472](https://www.ncbi.nlm.nih.gov/pubmed/15721472) |
| GSE20685 | 327 | GPL570 | [PMID: 21501481](https://www.ncbi.nlm.nih.gov/pubmed/21501481) |
| GSE20711 | 90 | GPL570 | [PMID: 21910250](https://www.ncbi.nlm.nih.gov/pubmed/21910250) |
| GSE21653 | 240 | GPL570 | [PMID: 20490655](https://www.ncbi.nlm.nih.gov/pubmed/20490655) |
| GSE22093 | 68 | GPL96 | [PMID: 21191116](https://www.ncbi.nlm.nih.gov/pubmed/21191116) |
| GSE2603 | 99 | GPL96 | [PMID: 16049480](https://www.ncbi.nlm.nih.gov/pubmed/16049480) |
| GSE26971 | 276 | GPL96 | [PMID: 21807638](https://www.ncbi.nlm.nih.gov/pubmed/21807638) |
| GSE29044 | 79 | GPL570 | [PMID: 23704896](https://www.ncbi.nlm.nih.gov/pubmed/23704896) |
| GSE2990 | 102 | GPL96 | [PMID: 16478745](https://www.ncbi.nlm.nih.gov/pubmed/16478745) |
| GSE31448 | 71 | GPL570 | [PMID: 22110708](https://www.ncbi.nlm.nih.gov/pubmed/22110708) |
| GSE31519 | 67 | GPL96 | [PMID: 21978456](https://www.ncbi.nlm.nih.gov/pubmed/21978456) |
| GSE32646 | 115 | GPL570 | [PMID: 22320227](https://www.ncbi.nlm.nih.gov/pubmed/22320227) |
| GSE3494 | 251 | GPL96, GPL97 | [PMID: 16141321](https://www.ncbi.nlm.nih.gov/pubmed/16141321) |
| GSE36771 | 107 | GPL570 | [PMID: 22564725](https://www.ncbi.nlm.nih.gov/pubmed/22564725) |
| GSE37946 | 41 | GPL96 | PMID: 22460789 |
| GSE41998 | 279 | GPL571 | [PMID: 23340299](https://www.ncbi.nlm.nih.gov/pubmed/23340299) |
| GSE42568 | 121 | GPL570 | [PMID: 23740839](https://www.ncbi.nlm.nih.gov/pubmed/23740839) |
| GSE43358 | 57 | GPL570 | [PMID: 25412710](https://www.ncbi.nlm.nih.gov/pubmed/25412710) |
| GSE43365 | 111 | GPL570 | no citation |
| GSE45255 | 139 | GPL96 | [PMID: 23618380](https://www.ncbi.nlm.nih.gov/pubmed/23618380) |
| GSE4611 | 153 | GPL96 | [PMID: 17317819](https://www.ncbi.nlm.nih.gov/pubmed/17317819) |
| GSE46184 | 74 | GPL96 | [PMID: 19054665](https://www.ncbi.nlm.nih.gov/pubmed/19054665) |
| GSE48390 | 81 | GPL570 | [PMID: 24098497](https://www.ncbi.nlm.nih.gov/pubmed/24098497) |
| GSE50948 | 156 | GPL570 | [PMID: 24443618](https://www.ncbi.nlm.nih.gov/pubmed/24443618) |
| GSE5327 | 58 | GPL96 | [PMID: 17420468](https://www.ncbi.nlm.nih.gov/pubmed/17420468) |
| GSE58812 | 107 | GPL570 | [PMID: 25887482](https://www.ncbi.nlm.nih.gov/pubmed/25887482) |
| GSE61304 | 62 | GPL570 | [PMID: 26474389](https://www.ncbi.nlm.nih.gov/pubmed/26474389) |
| GSE65194 | 164 | GPL570 | [PMID: 23144294](https://www.ncbi.nlm.nih.gov/pubmed/23144294) |
| GSE6532 | 82 | GPL96, GPL97, GPL570 | [PMID: 17401012](https://www.ncbi.nlm.nih.gov/pubmed/17401012) |
| GSE69031 | 130 | GPL571 | [PMID: 17157792](https://www.ncbi.nlm.nih.gov/pubmed/17157792) |
| GSE7390 | 198 | GPL96 | [PMID: 17545524](https://www.ncbi.nlm.nih.gov/pubmed/17545524) |
| GSE76275 | 265 | GPL570 | [PMID: 25208879](https://www.ncbi.nlm.nih.gov/pubmed/25208879) |
| GSE78958 | 424 | GPL571 | [PMID: 27148454](https://www.ncbi.nlm.nih.gov/pubmed/27148454) |
| GSE9195 | 77 | GPL570 | [PMID: 18498629](https://www.ncbi.nlm.nih.gov/pubmed/18498629) |

Table S2. *SPAG5* expression in all breast cancer patients with different subtypes

|  | | N | | Mean | | 95% CI | | Std. Error | | Std. Dev. | |
| --- | --- | --- | --- | --- | --- | --- | --- | --- | --- | --- | --- |
| ER status |  | |  | |  | |  | |  | |  |
| ER-  ER+  Entire sample | 2168 | | 602.64 | | 25.55 | | 13.03 | | 606.57 | |  |
|  | 3499 | | 434.48 | | 13.10 | | 6.68 | | 395.32 | |  |
|  | 5667 | | 498.81 | | 12.86 | | 6.56 | | 493.84 | |  |
| PR status  PR-  PR+  Entire sample  HER2 status  HER2-  HER2+  Entire sample  Lymph node status  LN-  LN+  Entire sample  Grade  1  2  3 | |  | |  | |  | |  | |  | |
|  |  | 1989 | | 572.13 | | 21.31 | | 10.87 | | 484.65 | |
|  |  | 1559 | | 442.90 | | 18.78 | | 9.57 | | 378.03 | |
|  |  | 3548 | | 515.35 | | 14.67 | | 7.48 | | 445.57 | |
|  |  |  | |  | |  | |  | |  | |
|  |  | 2682 | | 484.83 | | 14.58 | | 7.43 | | 385.01 | |
|  |  | 796 | | 690.89 | | 60.94 | | 31.05 | | 875.94 | |
|  |  | 3478 | | 531.99 | | 18.13 | | 9.24 | | 545.19 | |
|  |  |  | |  | |  | |  | |  | |
|  |  | 2829 | | 462.69 | | 15.50 | | 7.90 | | 420.33 | |
|  |  | 2165 | | 540.23 | | 24.98 | | 12.74 | | 592.67 | |
|  |  | 4994 | | 496.31 | | 13.98 | | 7.13 | | 503.77 | |
|  |  |  | |  | |  | |  | |  | |
|  |  | 576 | | 276.04 | | 15.05 | | 7.66 | | 183.96 | |
|  |  | 1795 | | 401.85 | | 16.97 | | 8.65 | | 366.53 | |
|  |  | 2058 | | 595.19 | | 20.98 | | 10.70 | | 485.23 | |
| Entire sample | | 4429 | | 475.33 | | 12.58 | | 6.41 | | 426.89 | |
| TP53 status | |  | |  | |  | |  | |  | |
| Wild-type | | 388 | | 402.12 | | 31.32 | | 15.93 | | 313.78 | |
| Mutated | | 272 | | 699.92 | | 87.15 | | 44.27 | | 730.08 | |
| Entire sample | | 660 | | 524.85 | | 41.76 | | 21.27 | | 546.38 | |
| Chemotherapy | |  | |  | |  | |  | |  | |
| Chemo-  Chemo+  Entire sample | | 1980  2295  4275 | | 402.86  531.63  471.99 | | 16.71  22.97  14.68 | | 8.52  11.71  7.49 | | 379.18  561.15  489.60 | |
| Endocrine therapy | |  | |  | |  | |  | |  | |
| Endo- | | 1766 | | 488.64 | | 21.12 | | 10.77 | | 452.62 | |
| Endo+ | | 1527 | | 402.79 | | 16.28 | | 8.30 | | 324.32 | |
| Entire sample | | 3293 | | 448.83 | | 13.69 | | 6.98 | | 400.54 | |

N: number of patients; CI: confidence interval; Std. Error: standard error; Std. Dev.: standard deviation; Chemo-: patients without chemotherapy; Chemo+: patients with chemotherapy; Endo-: patients without endocrine therapy; Endo+: patients with endocrine therapy.

Table S3. The comparison of gene expression level using Mann-Whitney U test or Kruskal-Wallis test

|  | N | Mean Rank | U | Z | P |
| --- | --- | --- | --- | --- | --- |
| ER status  ER-  ER+ |  |  |  |  |  |
|  | 2168  3499 | 3360.03  2508.07 | 4933351.00  2652481.00 | 19.05 | < 0.0001 |
| PR status  PR- | 1989 | 1963.93 | 1927197.50 |  |  |
| PR+ | 1559 | 1532.82 | 1173653.50 | -12.44 | < 0.0001 |
| HER2 status  HER2- | 2682 | 1680.69 | 909711.50 |  |  |
| HER2+ | 796 | 1937.65 | 1225160.50 | 6.34 | < 0.0001 |
| Lymph node status  LN- | 2829 | 2370.42 | 2702890.00 |  |  |
| LN+ | 2165 | 2663.55 | 3421895.00 | 7.12 | < 0.0001 |
| TP53 status  wild type | 388 | 265.69 | 27621.00 |  |  |
| mutated | 272 | 422.95 | 77915.00 | -10.43 | < 0.0001 |
| Chemotherapy |  |  |  |  |  |
| Chemo-  Chemo+ | 1980  2295 | 1893.57  2348.88 | 1788073.5  2756026.5 | -12.03 | < 0.0001 |
| Endocrine therapy |  |  |  |  |  |
| Endo- | 1766 | 1758.07 | 1544490 |  |  |
| Endo+ | 1527 | 1518.55 | 1152192 | -7.21 | < 0.0001 |

|  |  |  | H | Degrees of Freedom | P |
| --- | --- | --- | --- | --- | --- |
| Grade  1 | 576 | 1303.38 |  |  |  |
| 2 | 1795 | 1913.89 |  |  |  |
| 3 | 2058 | 2732.78 | 729.75 | 2.00 | < 0.0001 |

Chemo-: patients without chemotherapy; Chemo+: patients with chemotherapy; Endo-: patients without endocrine therapy; Endo+: patients with endocrine therapy.

Table S4. The comparison and correlation of SPAG5 with other markers of progression in assessing independent value

|  | RFS | | | OS | | | DMFS | | |
| --- | --- | --- | --- | --- | --- | --- | --- | --- | --- |
|  | n | HR | P-value | n | HR | P-value | n | HR | P-value |
| SPAG5 | 288 | 2.5 (1.36-4.6) | 0.0023 | 121 | 2.5 (0.86-7.24) | 0.081 | 141 | 2.09 (0.76-5.76) | 0.15 |
| P53 | 235 | 1.1 (0.59-2.03) | 0.77 | 121 | 0.62 (0.22-1.72) | 0.35 | 141 | 0.78 (0.27-2.23) | 0.64 |
| AURKA  BIRC5  MKI67 | 235 | 2.01 (1.05-3.84) | 0.031 | 121 | 1.91 (0.65-5.59) | 0.23 | 141 | 2.63 (0.84-8.26) | 0.086 |
|  | 235 | 1.51 (0.8-2.83) | 0.2 | 121 | 1.12 (0.42-3.03) | 0.82 | 141 | 1.44 (0.51-4.04) | 0.49 |
|  | 235 | 2.29 (1.19-4.42) | 0.011 | 121 | 1.32 (0.49-3.57) | 0.58 | 141 | 2.37 (0.82-6.85) | 0.1 |
| PCNA | 235 | 0.85 (0.46-1.56) | 0.59 | 121 | 1.91 (0.69-5.31) | 0.21 | 141 | 1.33 (0.48-3.68) | 0.59 |
| BUB1 | 235 | 2.08 (1.09-3.96) | 0.023 | 121 | 2.24 (0.75-6.71) | 0.14 | 141 | 3.56 (1.12-11.3) | 0.021 |
| TOP2A | 235 | 1.81 (0.95-3.47) | 0.069 | 121 | 1.76 (0.6-5.15) | 0.3 | 141 | 1.98 (0.68-5.79) | 0.2 |

RFS: Relapse free survival; OS: Overall survival; DMFS: Distant metastasis-free survival; HR: Hazard ratio

|  | Correlation coefficient | One-sided significance | |
| --- | --- | --- | --- |
| PCNA | 0.23 | <0.001 | |
| TOP2A | 0.64 | <0.001 | |
| TP53 | -0.08 | 0.001 | |
| BIRC5 | 0.6 | <0.001 | |
| AURKA | 0.69 | <0.001 | |
| BUB1 | 0.66 | <0.001 | |
|  | | |  |

Table S5. Subgroup analyses of *SPAG5* gene in association with RFS in ER+/- breast cancer subtype

| Subtypes | RFS (ER+) | | | RFS (ER-) | | | |
| --- | --- | --- | --- | --- | --- | --- | --- |
|  | n | HR | P-value | n | HR | P-value | |
| PR status  PR +  PR - | 514 | 2.06 (1.4-3.03) | 0.00017 | 23 | 3.13 (0.93-10.52) | 0.052 |  |
|  | 137 | 1.57 (0.85-2.92) | 0.15 | 346 | 1.35 (0.95-1.94) | 0.096 |  |
| HER2 status  HER2 +  HER2 - | 92 | 0.66 (0.28-1.55) | 0.34 | 92 | 0.89 (0.48-1.63) | 0.69 |  |
|  | 460 | 1.65 (1.13-2.43) | 0.0097 | 295 | 1.44 (0.98-2.1) | 0.059 |  |
| Lymph node status  Lymph node +  Lymph node - | 642 | 1.41 (1.06-1.87) | 0.017 | 227 | 1.1 (0.75-1.62) | 0.61 |  |
|  | 1126 | 1.86 (1.48-2.34) | <0.0001 | 378 | 0.89 (0.63-1.25) | 0.51 |  |
| Grade  1  2  3 | 288  653 | 2.5 (1.36-4.6)  1.93 (1.45-2.57) | 0.0023  <0.0001 | 28  73 | 3.98 (0.44-35.75)  1.38 (0.75-2.55) | 0.18  0.3 |  |
|  | 378 | 1.2 (0.87-1.67) | 0.27 | 293 | 1.06 (0.73-1.53) | 0.76 |  |
| TP53 status  Mutated  Wild type | 65 | 1.12 (0.53-2.38) | 0.77 | 66 | 0.48 (0.22-1.07) | 0.067 |  |
|  | 251 | 1.33 (0.86-2.06) | 0.2 | 29 | 4.07 (0.84-19.68) | 0.059 |  |

-: data not available
